# Effects of acupuncture and exercise on EEG characteristics and cognitive control in college students with high procrastination tendency

**DOI:** 10.3389/fpubh.2026.1725633

**Published:** 2026-04-02

**Authors:** Xinwang Chen, Yajing Guo, Ce Shi, Jing Wen, Yuan Gao, Lihua Wu, Haishui Jiang, Yongqi Yuan, Linze Wu, Huihui Yin, Yiming Wu

**Affiliations:** 1 College of Acupuncture-Moxibustion and Tuina, Henan University of Traditional Chinese Medicine, Zhengzhou, China; 2 College of the First Clinical Medicine, Anhui University of Chinese Medicine, Hefei, China; 3 College of Rehabilitation Medicine, Henan University of Traditional Chinese Medicine, Zhengzhou, China; 4 The Third Affiliated Hospital, Henan University of Traditional Chinese Medicine, Zhengzhou, China

**Keywords:** acupuncture, cognitive function, event-related potential, procrastination, university students

## Abstract

Procrastination is more than just a common human foible; it is a ubiquitous behavioral disorder that can negatively impact learning, productivity, and mental health. The psychological and neurological mechanisms underlying chronic procrastination are surprisingly complex, involving impairments in self-control, emotional regulation, and episode anticipation networks, and may lead to emotional distress, reduced self-confidence, and even physical symptoms that in turn can exacerbate procrastination. The aim of this proposed study is to investigate the clinical efficacy of acupuncture for university students with severe procrastination tendencies, to compare the efficacy of acupuncture to other potential interventions (Tai Chi and running exercise), and to examine the effects of acupuncture on cognitive functions related to procrastination. This parallel randomized controlled clinical trial will be conducted at the Third Affiliated Hospital of Henan University of Chinese Medicine, Henan Province, China. A total of ninety full-time college students will be randomly divided into three equal groups (acupuncture group, running group, and Tai Chi group). Each group will receive the assigned intervention for 30 min per session, 3 times per week on alternating weekdays, for a total of 4 weeks. The General Procrastination Scale (GPS) will serve as the primary outcome measure, while the Procrastination Assessment Scale for Students (PASS) and Physical Activity Rating Scale 3 (PARS-3) will be used as secondary outcome measures. Assessments using these outcome measures will be conducted at baseline (0 week) as well as after 2 and 4 weeks of intervention. Scalp electroencephalography (EEG) will be used to measure event-related potentials P300 and N200 during the Go/No-go Emotion Detection task and Task Initiation Delay Paradigm (TIDP) to investigate the impacts of these interventions on cognitive control and error detection abilities.

**Clinical trial registration:** https://www.chictr.org.cn/showprojEN.html?proj=278360, identifier: ChiCTR2500105950.

## Introduction

Procrastination, defined as “the behavior of voluntarily postponing planned tasks despite being able to anticipate harmful consequences” (1), is a highly prevalent behavioral disorder worldwide. Procrastination is particularly prevalent among college students, with more than 70% admitting to academic procrastination, of which about 16% exhibit serious academic procrastination (2). Another survey of 22,896 college students found that more than 90% reported procrastination habits, of which more than 60% indicated that this habit negatively affected learning. Procrastination behaviors are also common among college students in their daily lives, such as delaying the completion of daily tasks and decision-making. Procrastination manifests as postponement, indirect avoidance, and inefficiency, and can result in emotional distress, physical symptoms, and reduced self-confidence. For example, procrastinators often put off completing tasks, potentially missing deadlines, wait until the last minute to begin a task on the grounds that it is “not urgent” or they are “waiting for inspiration” (3), often resulting in suboptimal work. Even after beginning a task, many individual with procrastination tendencies may prioritize other unimportant activities such as scrolling through mobile phones or playing video games to avoid work or study that requires their full concentration. These frequent distractions reduce work efficiency and quality. Missed deadlines and poor work may in turn lead to self-blame and guilt, creating a cycle in which procrastination weakens self-efficacy and self-confidence, and results in even greater tendencies for task avoidance and delay. Procrastination impairs learning and work efficiency, and can lead to an increase in psychological distress and reduced quality of life. The persistence of such behaviors can lead to a loss of individual productivity, and incur a socio-economic burden, as the World Health Organization estimates that procrastination costs more than US $1.2 trillion globally (1).

Procrastination is associated with a variety of mental health problems, including depressive symptoms, anxiety, and higher stress levels (4), which may reduce quality of life (5) and disturb both digestive function and sleep (6, 7). In addition, procrastination may negatively impact physical health directly by reducing the frequency of healthy behaviors such as exercise and healthy eating.

Procrastination can be understood as a failure of self-regulation and explained by the interplay of four variables: the value associated with completing an activity, the expectation to achieve that value, the time remaining to reach the desired value, and level of impulsiveness (8). The capacity for starting challenging tasks is strongly dependent on activities of the self-control system, emotion regulation system, and episode anticipation system (9). The dorsolateral pre-frontal cortex (dlPFC) is considered a major hub of the self-control system responsible for top-down cognitive control (10). In brain models, the range of the self-control system has been extended to the anterior cingulate cortex (ACC). A large body of evidence indicates that the ACC is capable of conflict monitoring and error signal reinforcement learning during the decision-making process. Although it is challenging to directly detect or measure ACC functions in self-control, it plays a role in regulating cognitive resources that are beneficial to the self-control process (11). A neuroimaging study by Chen and colleagues found that procrastination tendency was positively correlated with gray matter volume (GMV) in the left insula, ACC, and parahippocampal gyrus (PHC), negatively correlated with dlPFC GMV and ACC gray matter density, and positively correlated with cortical thickness and cortical complexity of the bilateral orbitofrontal cortex (OFC) (9). The dlPFC and ACC are part of an integrated cognitive control network, and deficits in cognitive self-control have been linked to increased vulnerability for neuropsychiatric disorders. It is thus reasonable to speculate that this comprehensive self-control network is a core negative regulator of procrastination (12, 13).

Dysfunction of the emotion regulation network results in difficulty controlling and suppressing negative emotions such as anxiety and stress when facing tasks, which in turn can lead to procrastination. When individuals are unable to properly manage negative emotions, they may choose to avoid or delay tasks to temporarily relieve inner discomfort (14). The specific emotion regulation strategies employed by the individual can also influence procrastination behavior. Cognitive reappraisal strategies can help individuals view tasks from a positive perspective and reduce the generation of negative emotions, thereby reducing the tendency to procrastinate. On the other hand, expressive inhibition strategies only temporarily suppress emotional expression, but do not change the individual's cognition and emotional experience of the task, so any improvement in procrastination behavior will ultimately be limited (15). The emotional processes induced by task involvement are core factors influencing procrastination, so it is expected that procrastination behavior will be associated with the morphological properties and activity patterns of the emotional regulation network. Indeed, positive correlations were found between procrastination and GMV of insular lobes, GMD of the OFC, and complexity of the OFC (9).

The cognitive mechanisms underlying procrastination are well explained by emotion regulation theory. Simply stated, in order to avoid task-induced short-term negative emotions, individuals sacrifice long-term task rewards for more immediate positive experiences, leading to procrastination (12, 16). As a key subcortical region of the prominence network, the insula plays an important role in social emotions and disgust in response to task-evoked signals, and insular activity is strongly correlated with emotional downregulation and subsequent sequela. The function of the OFC is thought to be comparable to that of the insula, contributing to the regulation and further re-evaluation of negative emotions as part of the general emotion regulation network (17). The essence of procrastination is flawed intertemporal decision-making, which refers to an individual's choice after weighing “immediate satisfaction from not doing something now” against “future benefits from completing it later”. The core flaw lies in the evaluation of “the future”. Future evaluation is based on self-cognitive projections formed from past experiences, supported jointly by the parahippocampal cortex, which integrates past memories, and the ventromedial pre-frontal cortex, which simulates future events. An individual's expectations for the future directly influence procrastinatory behavior; vague or negative outlooks tend to promote procrastination while clear goals can reduce it. Moreover, the “phased future expectation” that breaks down long-term tasks into phased small goals can constrain the “temporal discounting effect”, a psychological bias where humans tend to underestimate long-term benefits. Thus, clarifying short-term benefits cane enhance long-term value, thereby reducing the urge to procrastinate (12, 18). The PHC is a central node of the medial temporal lobe (MTL) and is essential for episodic memory and episodic futuristic thinking. The ventromedial (vm) PFC also plays a role in episodic future thinking, especially in future-focused and goal-oriented anticipation (19, 20). While the MTL encodes the retrospective details of experiences, the vmPFC contributes to episodic future thinking by generating a more abstract imagination after the individual's own self-referential process. For this, the vmPFC makes use of updated self-knowledge (experience) simulations to deliberate on the process of future task costs (including emotional distress), outcomes (value), and work load. In this theoretical framework, one root cause of procrastination is a failure in belief related to imagining and evaluating the true future value of tasks, which is associated with structural abnormalities in the neural network responsible for envisioning future scenarios. Among these failures is a flaw in “episodic future thinking,” defined as the ability to imagine future task outcomes supported by the “episodic prospection network”. The core brain regions of this network include the parahippocampal cortex (PHC), which integrates past experiences to provide materials for imagining the future, and the ventromedial pre-frontal cortex (vmPFC), which assesses the value of future outcomes. Under normal circumstances, the two work synergistically to help individuals correctly recognize the long-term value of tasks and reduce procrastination. However, if the underlying circuitry is abnormal due to disruptions in cellular organization (or resultant abnormal morphology), the PHC may not provide a realistic basis for imagining the future, and the vmPFC may not accurately evaluate the future value of tasks. Ultimately, this impairment makes it difficult for individuals to perceive the long-term significance of tasks, leading to immediate task avoidance (18).

Acupuncture, a core intervention in this study, has its potential value for ameliorating procrastinatory behavior supported by modern neurophysiological research (21). We hypothesize that it exerts regulatory effects by targeting the core neural networks and psychological processes underlying procrastinatory behavior (9). Modern neurophysiological studies have fully confirmed that acupuncture can modulate the activity of key brain regions and neural networks associated with procrastination (22). Specifically, acupuncture stimulation at acupoints such as Baihui (GV20), Sishencong (EX-HN1), Neiguan (PC6), and Zusanli (ST36) can enhance the functional activity of the dorsolateral pre-frontal cortex (dlPFC) and anterior cingulate cortex (ACC)—the core nodes of the cognitive control network—thereby improving the top-down cognitive control, conflict monitoring, and error correction abilities that are deficient in individuals with high procrastination tendency (23). In addition, acupuncture can regulate the activity of the insula and orbitofrontal cortex (OFC) within the emotion regulation network, reduce the overactivation of the brain in response to negative emotional stimuli, and enhance individuals' ability to suppress and regulate negative emotions such as anxiety and stress when confronted with tasks, thus breaking the vicious cycle of emotional avoidance that triggers procrastination (24). Furthermore, acupuncture has been proven to promote neuroplasticity and improve the connectivity of the episodic prospection network consisting of the parahippocampal gyrus (PHC) and ventromedial pre-frontal cortex (vmPFC) (25). This effect helps optimize the brain's ability to envision future task outcomes and evaluate long-term values, thereby alleviating the intertemporal decision-making deficits that constitute the essence of procrastinatory behavior. Clinical and experimental studies have also confirmed that acupuncture can effectively improve attention, executive function, and impulse control in diverse populations, and these enhancements in cognitive functions are directly relevant to the correction of procrastinatory behavior (26).

The theory of planned behavior (TPB) is one of the most successful theoretical models for explaining the relationship between intention and behavior (including physical activities such as exercise), and can assist in elucidating the mechanism underlying the effects of physical activity on academic procrastination (27–29). The core propositions of the theory are that intentions can directly determine behavior, that behavioral attitudes, subjective norms, and perceived behavioral control are the three main predictors of behavioral intention intensity, and that the more positive an individual's attitude toward the goal, the stronger the subjective norm, the greater the perceived behavioral control, the stronger the intention to perform the goal-oriented behavior, and the more likely the individual is to participate in the behavior (i.e., without delay) (30, 31). Empirical studies have shown that TPB can explain nearly 40% of the variance in physical behavior, and all predictors of TPB can significantly predict the state of college students' participation in physical activity (32, 33). Thus, TPB provides a robust framework for procrastination research. A study examining procrastination behavior by college students and the efficacy of physical intervention based on the TPB framework concluded that physical activity has a negative predictive effect on procrastination behavior. As proposed by American psychologists Burka and Yuen in their book Procrastination: why you do it and what to do now (34, 35), physical activity can maintain an individual's physical health and enhance their courage to face postponed tasks, thereby reducing procrastination (36). In accord with this notion, Tao et al. reported a significant negative correlation between procrastination and physical activity among a cohort of 610 college students (37).

Tai Chi is a form of combined physical and cognitive training that may be particularly effective against procrastination. Tai Chi movement combined with cognitive training was reported to delay the progression of mild cognitive impairment (MCI) to dementia more effectively than cognitive training alone (38). Tai Chi combined with mindfulness training was also reported to enhance PFC activity and reduce decision hesitancy at the initiation of tasks (e.g., the “Should I start working or procrastinate” conflict). Moreover, Tai Chi with mindfulness training has also been found to improve Conners scale attention scores (*p* < 0.05) in parallel with the intensity of training (39, 40).

Event-related potentials (ERPs) are electroencephalographic waveforms reflecting specific neural processing events, including sensory perception, attentional allocation, and decision-making. Multiple studies have reported greater P200 amplitude by high procrastinators during the time delay and reward processing phases of delayed reward tasks, suggesting that these individuals allocate attentional resources more slowly and that motivationally driven attention to this information is elevated compared to low procrastination individuals (41, 42). In addition, while there was no significant difference in P300 amplitude between high and low procrastination individuals in the reward processing stage, the P300 amplitude of individuals with high procrastination was larger in the time delay processing stage, indicating higher cognitive load when processing time-delayed information. Alternatively, the P300 amplitude of individuals with low procrastination was larger during the reward processing phase, indicating a higher cognitive load when processing reward information (43, 44). Thus, the abnormal P300 component exhibited by high procrastination individuals indicates greater sensitivity to time delay in reward-based tasks, resulting in a greater propensity to choose smaller immediate rewards over greater delayed rewards, consistent with temporal motivation theory (TMT) (45, 46). This tendency may also be related to greater impulsivity.

The N200 ERP component usually appearing 160–240 ms after the presentation of a stimulus is primarily reflective of early attentional and cognitive processing, especially attentional allocation, and changes in N200 latency and amplitude are closely related to response inhibition and other aspects of cognitive control over behavior. Abnormalities in N200 among high procrastinators suggest a reduced ability to monitor task conflicts, making it difficult for these individuals to suppress distracting stimuli or prioritize tasks with perceived low short-term value (but possible higher delayed value) (47–49). One study reported a significant difference in N200 between the high and low procrastination groups as well as significant right lateralization of the visual ERP N1 among low procrastination individuals compared to the high procrastination group, suggesting that lower-level procrastinators are more alert to early information processing, while high procrastinators devote less attentional resources to tasks and show more impulsiveness and less control over immediate events (12). Patients with attention deficit hyperactivity disorder (ADHD) have also demonstrated a prolonged N200 latency, reflecting an inadequate allocation of attentional resources, and procrastination overlaps symptomatically with ADHD (including difficulty initiating tasks). Moreover, it is speculated that abnormalities in N200 may reflect similar underlying neural mechanisms (50, 51).

### Objectives

The purposes of the proposed study are to evaluate the clinical efficacy of acupuncture and exercise (running, Tai Chi) for reducing procrastination and to explore the underlying neurocognitive mechanisms by measuring ERP changes.

## Methods/design

### Study setting

**Figure 1:**
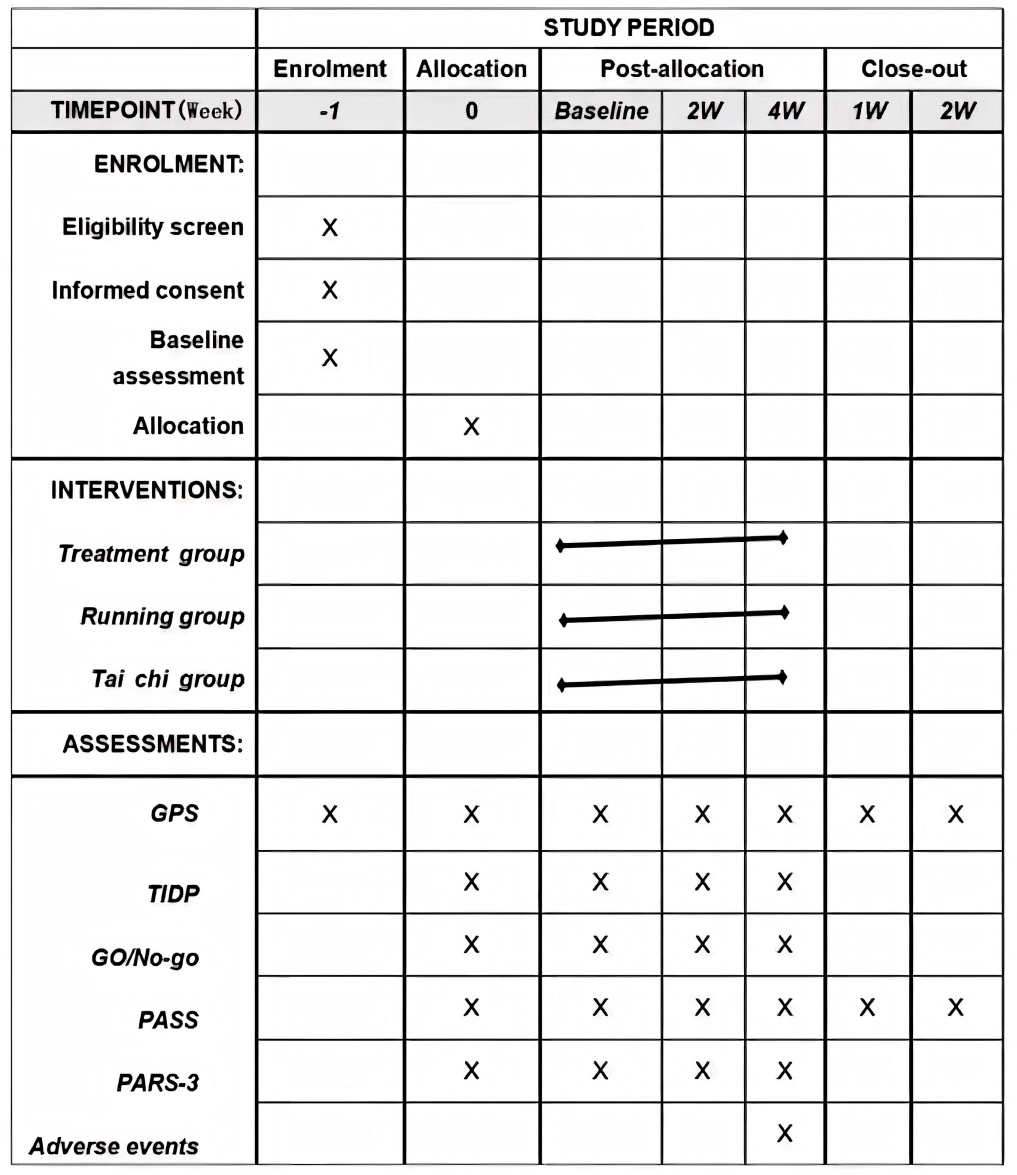
SPIRIT figure for schedule of enrollment, interventions, and assessments. Note: ‘ × ' indicates completed assessments. TIDP, task initiation delay paradigm; GO/No-go, Go/No-go emotional detection paradigm; ERPs, event-related potentials; GPS, general procrastination scale; PASS, procrastination assessment scale for students; PARS-3, physical activity rating scale-3.

This parallel randomized controlled clinical trial will be conducted at Third Affiliated Hospital of Henan University of Chinese Medicine, Henan Province, China, according to the principles outlined in Consolidated Standards of Reporting Trials (CONSORT), Standards for Reporting Interventions in Acupuncture Clinical Trials (STRICTA), and Standard Protocol Items: recommendations for Interventional Trials (SPIRIT) guidelines (Figure 1). Ninety full-time college students will be randomly and equally assigned to acupuncture, running, and Tai Chi groups. The allocated intervention will be conducted for 30 min, 3 times per week on alternating weekdays for a total of 4 weeks (w). The General Procrastination Scale (GPS) will be used as the primary outcome measure, with the Procrastination Assessment Scale for Students (PASS) and Physical Activity Rating Scale 3 (PARS-3) used as secondary outcome measures. Outcome measures will be conducted at baseline (0 w) and after 2 and 4 weeks of intervention. In addition, P300 and N200 will be measured by scalp EEG during the Go/No-go Emotion Detection task and Task Initiation Delay Paradigm (TIDP) to observe the effects of these interventions on cognitive control and error detection abilities.

### Recruitment

The study will recruit participants with procrastination from the Dongming Road Campus of Henan University of Traditional Chinese Medicine through posters stickers and online advertisements. Candidates who meet the inclusion criteria, do not meet any exclusion criteria, and agree to participate by providing written informed consent will be enrolled until the preset maximum sample size (n = 90) is reached. Additionally, 30 healthy subjects will be recruited as controls. The healthy control group is included to provide normative behavioral and electrophysiological reference values, rather than to serve as a treatment control for intervention effects.

#### Inclusion criteria

Preset study inclusion criteria are as follows:

1. Full-time university student;
2. GPS score ≥ 70 points;
3. No psychotropic drug intake or related acupuncture treatment within the past 30 days;
4. Providing informed consent.

#### Exclusion criteria

The following exclusion criteria will be applied during enrolment:

1. Organic mental disorders, diseases related to surgical history, and current use of psychoactive substances;
2. Family history of other mental illnesses such as dementia, schizophrenia, mania, and addiction;
3. Impaired consciousness or agitation, or otherwise unable to cooperate with task completion or EEG collection;
4. Skin lesions or skin diseases.

#### Dropout criteria

In addition, we will preset dropout criteria as follows:

1. Mistaken admission (subsequently found not to meet one or more inclusion criteria or found to meet one or more exclusion criteria);
2. Poor compliance or voluntary withdrawal;
3. Onset of treatments prohibited by this protocol, or changing treatment methods during the program;
4. Serious adverse reactions to the interventions.

##### Dropped cases will be handled as follows:

1. After drop out, the attending doctor will try to contact the subject by phone or letter to obtain the reason(s) and schedule a final visit if possible to complete the assessable items.
2. For cases that quit the trial due to adverse reactions or ineffective treatment, the attending doctor will take appropriate measures in accordance with current treatment guidelines.
3. The “Summary of Treatment Completion” and “Clinical Trial Completion” in the CRF will be filled in.
4. All excluded and dropped cases will be included in the intention-to-treat analysis after the trial ends.

### Randomization

Participants will be allocated to the three intervention groups based on random numbers generated using SPSS 21.0. Allocation numbers will be sealed in opaque envelopes labeled with a serial number. During the clinical implementation, the order in which eligible cases are included in the trial will correspond to the serial number on the envelope, and the envelopes will be opened in order and participants grouped according to the prompts on random number cards.

### Blinding

Blinding of participants to group allocation is not possible due to the active nature of the interventions. Therefore, we will institute three-way blinding of group allocation among administers of psychological assessments, recorders of electroencephalographic data, and operators responsible for subsequent analyses.

### Intervention

#### Acupuncture group

All acupuncture practitioners participating in this study are licensed traditional Chinese medicine acupuncture therapists with at least 5 years of clinical experience. They will receive uniform training to master the study protocol and thereby reduce treatment variation. Based on our clinical experience, the following acupoints will be selected for this study: Baihui (GV20), Sishencong (EX-HN1), Zusanli (ST36), and Neiguan (PC6). Acupoint positioning will be conducted according to the 2006 National Standard of the People's Republic of China (GB/T 12346-2006) “Acupoint Name and Positioning” as follows: Baihui (GV20), 5 inches below the hairline; Sishen cong Acupoint (EX-HN1), 4 acupoints located 1 inch to the left and right of the Baihui Acupoint; Zusanli acupoint (ST36), located on the anterolateral side of the Dubi acupoint (ST35), 3 inches below the Dubi and one transverse finger width outside the anterior tibial crest; Neiguan (PC6): in the forearm area, 2 inches above the transverse stria of the carpal palmes, between the palmaris longus tendon and the flexor carpi radialis tendon.

Acupuncture operation will be conducted according to “Acupuncture and Moxibustion” edited by Liang FanRong, the textbook of the 13th 5-Year Plan. Briefly, the participant will be positioned in the sitting or supine position. After routine skin disinfection, φ0.25 mm × 25 mm stainless steel needles will be inserted, and left in position for 30 min. Needles removal and post-intervention care will follow standard institutional safety procedures.

Compliance is defined as an attendance rate ≥80% (≥9 sessions completed over 4 weeks). Participants with attendance < 80% will not be excluded from the ITT analysis but will be excluded from the PP analysis.

#### Running group

The running group will perform moderate-intensity continuous training (MICT) according to American College of Sports Medicine (ACSM) guidelines with precise exercise intensity control according to heart rate reserve (HRR) calculations. Maximum heart rate (HRmax) and anaerobic threshold heart rate (ATHR) will be measured for each participant at baseline using a standard treadmill escalating load test (Bruce protocol), and HRR calculated as HRR = HRmax-Resting Heart Rate (RHR). Training intensity will be maintained in the 60–75% HRR range (equivalent to 70–85% of maximum heart rate) by real-time monitoring using a wearable heart rate monitor. Participants will run 3 times per week for 30 min, including 5 min of brisk warm-up walking (heart rate controlled at 40–50% of HRR), 20 min of target intensity running, and 5 min of cold walking and dynamic stretching. The training time will be fixed at 17:00–18:30 every day to reduce the impacts of circadian factors. The dual-track “check-in + sports data upload” system will be used to supervise the activity, and participants will wear sports bracelets to record running distance, pace, and heart rate data. These data will be submitted weekly. Compliance is defined as an attendance rate ≥80% (≥9 sessions completed over 4 weeks). Participants with attendance < 80% will not be excluded from the intention-to-treat (ITT) analysis but will be excluded from the per-protocol (PP) analysis.

#### Tai Chi group

The simplified 24-style Tai Chi will be used as a second intervention exercise as it is concise and easy to master by novices while retaining the core essentials of traditional Tai Chi. It consists of 24 movements, such as “starting momentum”, “wild horse mane”, and “white crane wings”, with a drill duration of about 8–10 min, and a total session duration of about 30 min including warm-up, practice, and the closing posture of Tai Chi. Training will be delivered 3 times per week at 17:00–18:30 both for convenience and temporal consistency with the other interventions. Each training session will be divided into three phases: a 5-min warm-up (including neck rotation, joint movement, lunge leg press), 20-min Tai Chi routine practice (including two repetitions), and the 5-min closing (pranayama relaxation, tapping the meridians). The selected Tai Chi training intensity aims for “slight sweating, steady breathing, and consistent movements”, and will be monitored by the Subjective Fatigue Scale (RPE), which requires the participants to maintain an RPE score of 11–13 (slightly tired but sustainable) during the training. At the same time, participants will use a wearable device to ensure that average heart rate remains at 50–65% of HRmax. The Tai Chi intervention will adopt “online + offline” dual supervision. During offline training, the coach will observe the participants' movements on video submitted via an online video application, from which the coach will provide remote guidance. Attendance will be summarized monthly. Compliance is defined as an attendance rate ≥80% (≥9 sessions completed over 4 weeks); if attendance is < 80%, reasons will be documented and participants will be encouraged to improve, with reasons recorded for sensitivity analysis.

### Measurement of event-related potentials by EEG

A total of 120 participants will be recruited, including 90 with high levels of procrastination to be allocated randomly and equally to acupuncture, running, and Tai Chi groups, and a healthy control group (n = 30). All members of the research team received systematic training in EEG data acquisition and analysis, and participated in the full workflow of the pilot study, including data collection and pre-processing. EEG data will be recorded using a 32-channel actiCHamp Plus system (Brain Products, Germany). Electrodes will be placed according to the international 10–20 system, with bilateral mastoids serving as reference electrode points. Electrode impedances will be maintained below 5 kΩ, and signals will be sampled at 500 Hz. Online filtering will include a high-pass filter (0.1–0.5 Hz), a low-pass filter (30–50 Hz), and a 50 Hz notch filter to eliminate line noise. Data analyses will focus on midline frontal (Fz), central (Cz), parietal (Pz), and lateral frontal (F3, F4) electrodes. Pre-processing procedures, including filtering, artifact rejection, and independent component analysis (ICA), will be performed using MATLAB R2020b and EEGLAB v2019.0.

#### Experimental paradigms

##### Go/no-go emotional detection task

The Go/No-go emotional detection paradigm will be used to assess participants' emotional attentional bias and cognitive control. Stimuli will consist of 50 pictures, distributed equally across five emotional categories: anger, fear, happiness, sadness, and neutral (10 images per category). Stimuli will be presented in five pseudo-randomized blocks to avoid consecutive repetition of the same emotional category. In the task design, Go trials (25%) will only involve neutral images; participants will be required to respond quickly and accurately by pressing the spacebar when a neutral image is presented. No-go trials (75%) will only involve emotional images (depicting anger, fear, happiness, or sadness) and require participants to withhold all responses (no neutral images will be included in No-go trials). Each trial will begin with a central fixation cross (“+”), which will remain until stable fixation is established. The fixation cross will then be replaced by a stimulus (either emotional or neutral) presented for 150 ms, followed by a variable inter-stimulus interval (ISI) of 0–500 ms. Before the formal task, participants will complete 10–15 practice trials to ensure understanding of task requirements.

##### Task initiation delay paradigm (TIDP)

The Task Initiation Delay Paradigm (TIDP) will be used to evaluate participants' readiness for task initiation and their perception of time delay. Stimuli will consist of a green fixation cross (“+”) serving as a preparatory cue and target stimuli represented by the letters “X” or “O.” Three delay conditions will be introduced between the cue and target stimulus: 100 ms, 500 ms, and 2,000 ms. Ten trials will be conducted under each condition in a total of four randomized blocks. Participants will be instructed to respond as quickly as possible by pressing the “F” key when “X” appears and the “J” key when “O” appears. The trial sequence will proceed as follows. A fixation cross will be presented at the center of the screen until participants are prepared. A green cue (“+”) will then appear for 100 ms, followed by a blank screen representing the delay interval (100, 500, or 2,000 ms). After the delay, the target stimulus will appear and remain visible until the participant responds or for a maximum of 2,000 ms. Each trial will be followed by a 500 ms inter-trial interval (ITI). Prior to the formal task, participants will complete 5–10 practice trials to familiarize themselves with the delay conditions and response rules.

### Expected results

**Figure 2:**
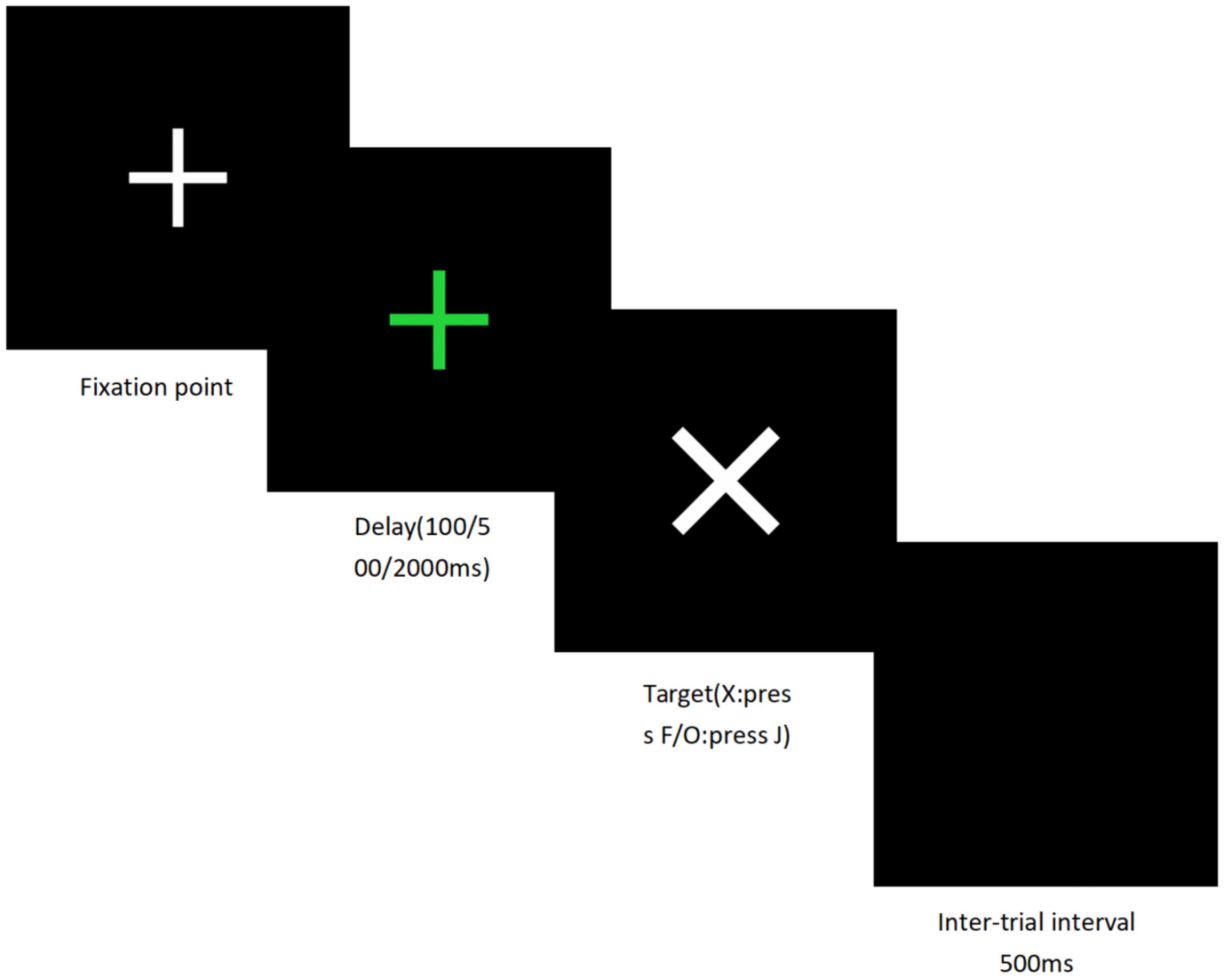
Task initiation delay paradigm (TIDP) process diagram. It depicts the process of the task initiation delay paradigm. It starts with a fixation point, followed by a delay period (100/500/2,000 ms) with a green cross. Then a target appears (press “s” for “X”, press “j” for “O”), and finally, there is a 500 ms inter-trial interval.

It is hypothesized that significant differences in ERP components will be observed between high-procrastination individuals and healthy controls, and that these components will be modulated by the interventions. In the Go/No-go task, high-procrastination participants are expected to show reduced N200 amplitudes and prolonged latencies, whereas healthy controls will display normal N200 amplitudes with shorter latencies. The high-procrastination participants are also anticipated to exhibit greater P300 amplitudes in response to negative emotional stimuli, while healthy controls are expected to show greater amplitudes during neutral (Go) trials. Following acupuncture or exercise interventions, N200 amplitudes are expected to increase toward the levels observed in healthy controls, accompanied by shortened latencies. Alternatively, P300 amplitudes during negative emotional processing are expected to decrease, whereas amplitudes during neutral (Go) trials are expected to increase, reflecting a reduction in emotional attentional bias and an enhancement of task-focused processing. In the TIDP, the P300 amplitude during long-delay trials (2,000 ms) is expected to decrease after the intervention, indicating a reduced cognitive load when perceiving temporal delays, greater tolerance for “delayed gratification”, and attenuated impulsive procrastination tendencies. Collectively, these ERP modulations may serve as objective neurophysiological markers for evaluating the effectiveness of interventions aimed at improving cognitive functioning among individuals with high procrastination tendencies. Prior to the formal task, participants will complete 5–10 practice trials to familiarize themselves with the delay conditions and response rules. The specific process of this task is illustrated in Figure 2 (Figure 2: task initiation delay paradigm (TIDP) process diagram).

### Healthy control group

The healthy control group will not receive any intervention. Both GPS/PASS scales and ERP (P300, N200) under Go/No-go emotion task and TIDP will be collected at baseline”.

## Outcomes

### Primary outcome

General Procrastination Scale (GPS) will be used as the primary outcome measure. The GPS is one of the most widely used and validated trait procrastination scales and has demonstrated good applicability for Chinese college students. It consists of 20 items scored on a 5-point Likert scale from “strongly disagree” (1 point) to “strongly agree” (5 points), and covers multiple domains including academic activities and activities of daily living (52). Note that higher scores on certain items are indicative of greater procrastination while higher scores on others are indicative of lower procrastination tendency, so some item scores are reversed before summation. After appropriate reversal, a higher total score is indicative of greater procrastination tendency, with a score between 20 and 49 points indicating low, 50–69 moderate, and 70–100 high procrastination. Xue and colleagues reported a Cronbach's α coefficient of 0.807 and a test–retest correlation coefficient of 0.756 after 4 weeks, indicating good internal consistency and reliability (53). Exploratory and confirmatory factor analysis revealed a two-factor structure consisting of active avoidance and lack of planning, and the model fitting effect was good (CFI=0.914, TLI=0.901, RMSEA=0.069, SRMR = 0.072). The GPS was also positively correlated with total scores on the Irrational Procrastination Scale and the Academic Burnout Scale (r = 0.753, 0.677, all *P* < 0.001), supporting good reliability and validity (54).

### Secondary outcomes

The Procrastination Assessment Scale for Students (PASS) was developed by Solomon and Rothblum in 1984 to measure students' academic procrastination behavior, including for coursework, test preparation, and essay writing (55). In the proposed study, the first part of the scale, revised by Hou and Gai (56), will be used as a secondary outcome measure. This section consists of 18 items covering 6 tasks: “writing term papers”, “reviewing exam preparations”, “academic assignments assigned by teachers”, “academic administration tasks”, “attendance assignments”, and “general school activities”. All items are scored on a 5-point Likert scale, and the scores of the first two items related to each of the six tasks are added together to calculate 6 task subscores from 2 to 10. The total score ranges from 12–60 points, with a higher score indicating a higher degree of academic procrastination. A total score >36 indicates problematic academic delay, while a subscore > 6 indicates procrastination on a specific task. In the proposed study, a score of 24 will be defined as severe procrastination. A previous study reported a Cronbach's α coefficient of 0.82 and test–retest coefficient of 0.78 after 2 weeks, indicating good internal consistency and reliability. Exploratory and confirmatory factor analysis demonstrated a three-factor structure, and model fitting was good (CFI = 0.921, TLI = 0.912, RMSEA = 0.071, SRMR = 0.068). Further, Yip and colleagues reported that the PASS score was significantly correlated with both the GPS score and academic performance (*r* = 0.65, 0.45, all *P*% 3C0.001), supporting good construct validity (55).

### Adverse events

**Figure 3:**
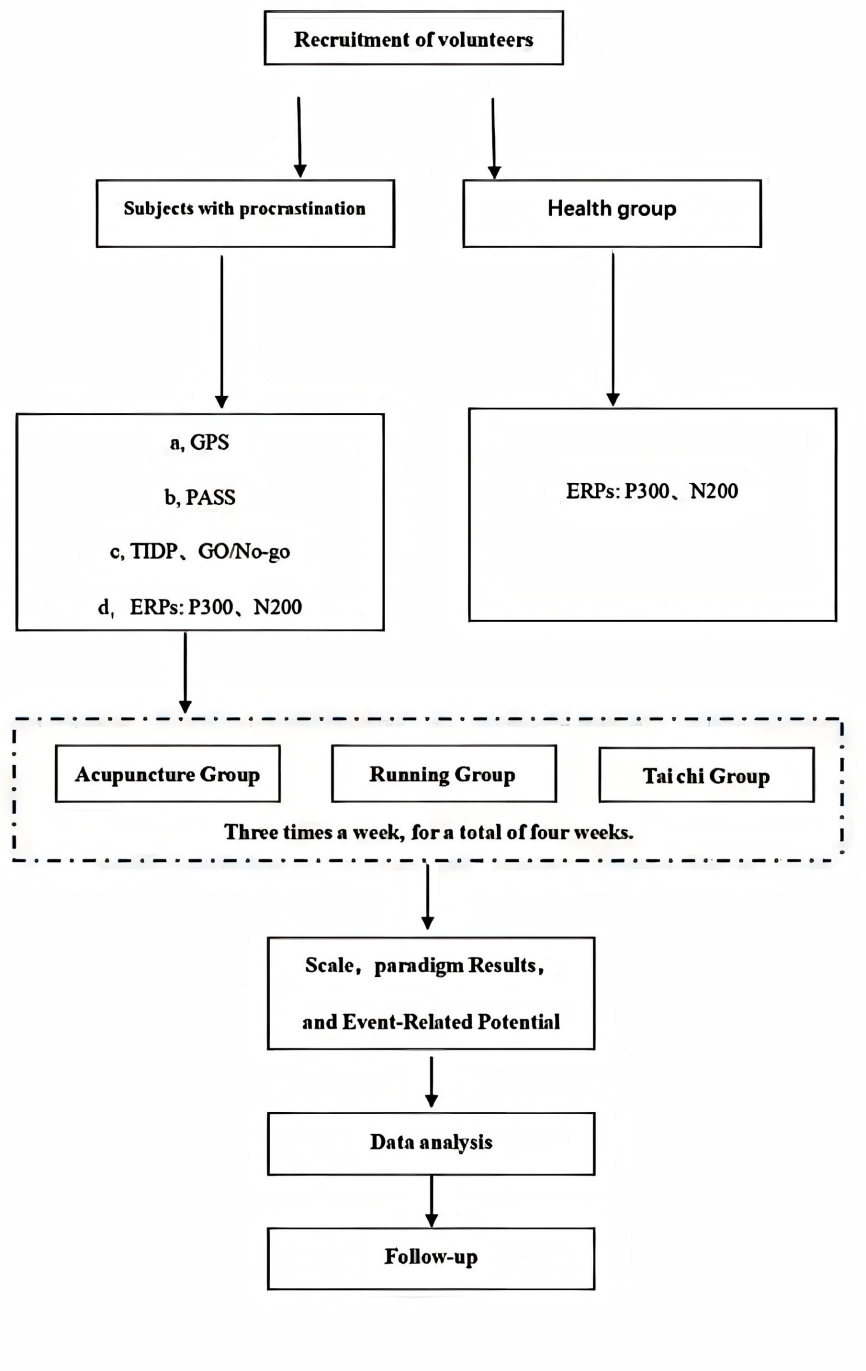
Schematic of the trial protocol. The primary outcome measure will be the GPS and secondary outcome measures will include the PASS. In addition to baseline, intervention, and immediate post- intervention measures, follow-up will be conducted in the first and second weeks after completion of the treatment plan.

Adverse events that may occur during acupuncture include broken, syncope, local hematoma, infection, and abscess, while post-acupuncture discomforts may include residual acupoint site pain, nausea, vomiting, palpitations, dizziness, headache, anorexia, and insomnia. Timely recording of these events is critical to enhance participant safety. In the proposed study, the incidence rates and average frequencies of these events will be recoded on a specific form for review and appropriate follow-up (Figure 3).

### Follow-up

Follow-up will be conducted in the first and second weeks after completion of the treatment plan (Figure 2).

#### Sample size

Sample size estimation was informed by pilot data and by consideration of realistic and conservative effect size assumptions appropriate for behavioral intervention studies. In a pilot study, post-intervention General Procrastination Scale (GPS) scores were 32.5 ± 6.8 in the acupuncture group, 38.2 ± 7.2 in the running group, and 41.6 ± 6.5 in the Tai Chi group after 8 weeks of intervention. Importantly, similar between-group trends were already observable at 4 weeks, supporting the feasibility of a 4-week intervention in the present trial.

However, given the exploratory nature of the current study and the risk of overestimating effects based on pilot data, sample size calculation for the main trial was performed assuming a conservative medium-to-large effect size. Using G^*^Power 3.1, a one-way analysis of variance (ANOVA) design with three intervention groups was specified, with a two-sided significance level of α = 0.05 and statistical power (1–β) = 0.90. An effect size of Cohen's *f* = 0.40 was selected, consistent with effect sizes commonly reported in behavioral and non-pharmacological intervention studies targeting self-regulation and cognitive control outcomes.

Under these assumptions, the required sample size was estimated to be 27 participants per group. Allowing for an anticipated dropout rate of approximately 10%, the final target sample size was set at 30 participants per intervention group, resulting in a total of 90 participants with high procrastination tendencies. In addition, 30 healthy control participants without procrastination will be recruited to provide normative behavioral and electrophysiological reference values.

### Data management and monitoring

Outcome measures, adverse events, and safety assessment data will be recorded systematically at indicated timepoints on case report forms (CRFs). A dedicated outcome evaluator will ensure the timeliness, accuracy, and completeness of data entry in accordance with requirements of the International Common Reporting Format (ICH-GCP E3/E6 standard). All paper and electronic records will be kept for at least 5 years after the end of the study to meet the traceability requirements of regulatory agencies. The anonymized trial data will be available to peer researchers for at least 3 years after the publication of the first main article, but a patient privacy protection protocol will be strictly implemented throughout, and all personally identifiable information (including name, ID number, contact number, etc.) will be encoded and encrypted to ensure that the data can be desensitized before it can be included in the analysis. The research protocol presented here has been independently reviewed and optimized by experts in the fields of acupuncture, emergency medicine, methodology, and biostatistics to ensure scientific and ethical integrity. The Institutional Ethics Committee (IEC) of the Third Affiliated Hospital of Henan University of Traditional Chinese Medicine will conduct annual on-site verification and data quality assessment independently of the investigators and the sponsor, and any changes in operating procedures shall be recorded on a Protocol Deviation Report Form and submitted to the Ethics Committee and the China Clinical Trial Registry for filing and review.

### Data analysis

The primary analysis will be conducted using a linear mixed-effects model, with fixed effects for group (acupuncture, running, Tai Chi), time (baseline, week 2, week 4), and their interaction, and a random intercept for participants to account for within-subject correlation due to repeated measurements. Baseline GPS score, age, sex, and baseline ERP indices will be included as covariates to control for inter-individual variability in procrastination-related traits.

This mixed-effects modeling framework constitutes the primary analytic strategy and will be implemented under the intention-to-treat (ITT) principle, allowing inclusion of all available observations without imputing missing values, under a missing-at-random assumption.

Prior to model fitting, distributional characteristics of continuous variables will be assessed using the Shapiro–Wilk test. Descriptive comparisons of baseline demographic and clinical characteristics will be performed using analysis of variance (ANOVA) or the Kruskal–Wallis test for continuous variables, and chi-square or Fisher's exact tests for categorical variables, as appropriate, to ensure baseline comparability between groups.

Secondary and exploratory analyses may include pairwise comparisons or non-parametric tests (e.g., Mann–Whitney *U*-test or Wilcoxon signed-rank test) where appropriate for specific outcomes or sensitivity checks. For outcomes with repeated measurements that meet parametric assumptions, repeated-measures ANOVA may be used as a supplementary analysis; otherwise, non-parametric alternatives such as the Friedman test will be applied.

Sensitivity analyses will be conducted to evaluate the robustness of the primary findings, including per-protocol analyses restricted to participants with adherence ≥80% and models incorporating adherence (number of completed sessions) as a continuous covariate.

For secondary outcomes and exploratory ERP analyses involving multiple indices or task conditions, false discovery rate (FDR) correction will be applied within pre-defined families of related comparisons. Statistical significance will be defined as a two-sided *P*-value < 0.05. All analyses will be performed using PASS 18.0, with statistical consultation provided by an institutional biostatistician.

### Ethics and dissemination

This study involves human participants. All research protocols have been reviewed and approved by the Institutional Ethics Committee (IEC) of the Third Affiliated Hospital of Henan University of Traditional Chinese Medicine (approval number: 2025HL-017) before the study commences. In accordance with the Declaration of Helsinki and the requirements of the Good Practice for Clinical Research of Drugs, subjects will provide written informed consent after a full explanation of study aims, potential adverse events, and rights, including the right to withdraw from the study at any time without explanation.

## Discussion

The proposed study aims to evaluate the potential therapeutic effects of acupuncture and exercise (running and Tai Chi) on procrastination among college students and to elucidate the underlying cognitive mechanisms. Procrastination is a complex behavioral disorder involving multiple neural networks regulating self-control, emotion, and motivation by reward. The study design fully considers the multidimensional neural mechanisms of procrastination by measuring electrophysiological biomarkers reflecting cognitive control, attention sharing, and error detection ability (such as the ERPs P300 and N200) in addition to psychometric scale scores. It is anticipated that these multimodal measurements will provide a more comprehensive understanding of the neural mechanisms underlying procrastination, identify the most appropriate evidence-based interventions, and reveal potential biomarkers for future mechanistic and clinical investigation.

Acupuncture and exercise have numerous advantages as non-pharmacological interventions for neuromodulation and physiological regulation, respectively. Acupuncture may improve procrastination behavior by modulating brain self-control networks (including the dlPFC and ACC) and emotion regulation networks (involving the insula and OFC), while exercise may further enhance the effects of acupuncture-induced neuromodulation by promoting neuroplasticity and improving emotional regulation. The study design provides a comprehensive framework for exploring the efficacy of different interventions (acupuncture vs. running and Tai Chi) and a foundation for future studies testing combined interventions for the non-pharmaceutical treatment of procrastination.

This RCT will strictly follow the CONSORT and STRICTA guidelines to ensure scientific rigor. The sample size was set based on statistical principles, and is sufficient to compensate for participant attrition, which is expected to be low given that the interventions are non-invasive, safe, and scheduled for convenience. Further, the proposed measurements were selected based on extensive research describing the neurological control impairments contributing to procrastination. For instance, acupoint selection (Baihui, Sishencong, Zusanli, Neiguan) is based on traditional Chinese medicine theories and these targets are documented to regulate self-control and emotion. Further, the exercise interventions (running and Tai Chi) have been studied extensively for physiological and psychological effects, and intensity will be preciselycontrolled through heart rate monitoring. The effects of these interventions on procrastination will also be assessed using multiple psychometric scales (the GPS and PASS) and ERPs (e.g., P300 and N200) reflective of cognitive control and emotion regulation for comprehensive evaluation at behavioral and neurophysiological levels.

Nonetheless, there are potential limitations and challenges. First, study participants will be college students to reduce possible response heterogeneity due to age, general health, and education among other factors. However, applicability of the results to other populations will require additional study. Future studies should extend recruitment to people of different ages and occupations to verify the generalizability of the findings. Second, the planned follow-up period is relatively short as both data acquisition and analyses will be labor-intensive. However, it may still be possible to schedule later follow-up dates. Future studies could also set longer follow-up periods to assess the longevity of these interventions and factors associated with recurrence. In addition, the study will not examine the broad physiological and psychological impacts of acupuncture and exercise, and will only indirectly assess the changes in neural networks through ERP indicators. Future research combining functional magnetic resonance imaging (fMRI) and other techniques is warranted to further reveal the cellular and circuit-level effects of acupuncture and exercise on brain networks relevant to procrastination.

This study will comprehensively assess the therapeutic effects of acupuncture and exercise on the EEG characteristics and cognitive control mechanisms of college students with high procrastination levels by combining multiple psychometric instruments and task-dependent ERP measures. The research design is based on extensive literature reviews and is of sufficient scale to detect statistically significant and reproducible outcomes. Future studies can further validate this study by expanding the sample size, extending the follow-up time, and combining multiple neuroimaging techniques.

## References

[1] Steinert C Heim N Leichsenring F. Procrastination, perfectionism, and other work-related mental problems: prevalence, types, assessment, and treatment-a scoping review. Front Psychiatry. (2021) 12:736776. doi: 10.3389/fpsyt.2021.736776 34707522 PMC8542725

[2] Yang CG Guo Z Zheng YL Hou HF. Analysis of procrastination phenomenon among college students. Med Inform. (2019) 32:32–34.

[3] Johansson F Rozental A Edlund K Grotle M Rudman A Jensen I . Trajectories of procrastination among Swedish University students over one academic year: a cohort study. BMC Psychology. (2024) 12:559. doi: 10.1186/s40359-024-02072-2 39407255 PMC11481787

[4] Van Wendelien E. A meta-analytically derived nomological network of procrastination. Pers Individ Diff . (2003) 35:1401–18. doi: 10.1016/S0191-8869(02)00358-6

[5] Alexander Rozental Erik Forsell Andreas Svensson . Psychometric evaluation of the Swedish version of the pure procrastination scale, the irrational procrastination scale, and the susceptibility to temptation scale in a clinical population. BMC Psychology. (2014) 2:54. doi: 10.1186/s40359-014-0054-z 25566392 PMC4269972

[6] Przepiórka A Błachnio A Siu NY. The relationships between self-efficacy, self-control, chronotype, procrastination and sleep problems in young adults. Chronobiol Int. (2019) 36:1025–35. doi: 10.1080/07420528.2019.1607370 31070062

[7] Ma X Meng D Zhu L Xu H Guo J Yang L . Bedtime procrastination predicts the prevalence and severity of poor sleep quality of Chinese undergraduate students. J Am Coll Health. (2022) 70:1104–11. doi: 10.1080/07448481.2020.1785474 32669056

[8] Steel P Koenig CJ. Integrating theories of motivation. Acad Manag Rev. (2006) 31:889–913. doi: 10.5465/amr.2006.22527462

[9] Chen Z Liu P Zhang C Feng T. Brain morphological dynamics of procrastination: the crucial role of the self-control, emotional, and episodic prospection network. Cereb Cortex. (2020) 30:2834–53. doi: 10.1093/cercor/bhz278 31845748

[10] Hu Y Liu P Guo Y Feng T. The neural substrates of procrastination: a voxel-based morphometry study. Brain Cogn. (2018) 121:11–6. doi: 10.1016/j.bandc.2018.01.001 29309854

[11] Holroyd CB Coles MGH. The neural basis of human error processing: reinforcement learning, dopamine, and the error-related negativity. Psychol Rev. (2002) 109:679–709. doi: 10.1037/0033-295X.109.4.679 12374324

[12] Zhang R Chen Z Feng T. The triple psychological and neural bases underlying procrastination: Evidence based on a two-year longitudinal study. Neuroimage. (2023) 283:120443. doi: 10.1016/j.neuroimage.2023.120443 37925799

[13] Chen Z Feng T. Neural connectome features of procrastination: current progress and future direction. Brain Cogn. (2022) 161:105882. doi: 10.1016/j.bandc.2022.105882 35679698

[14] Guo XD Zheng H Ruan D Hu DD Wang Y Wang YY . Cognitive and affective empathy and negative emotions: The mechanism of emotion regulation. Acta Psychol Sin. (2023) 55:892–904. doi: 10.3724/SP.J.1041.2023.00892

[15] Gross JJ John OP. Individual differences in two emotion regulation processes: implications for affect, relationships, and wellbeing. J Pers Soc Psychol. (2003) 85:348–62. doi: 10.1037/0022-3514.85.2.348 12916575

[16] Li K Zhang R Feng T. Functional connectivity in procrastination and emotion regulation. Brain Cogn. (2024) 182:106240. doi: 10.1016/j.bandc.2024.106240 39515273

[17] Wang H-Y You H-L Song C-L Zhou L Wang S-Y Li X-L . Shared and distinct prefrontal cortex alterations of implicit emotion regulation in depression and anxiety: An fNIRS investigation. J Affect Disord. (2024) 354:126–35. doi: 10.1016/j.jad.2024.03.032 38479517

[18] Zhao X Zhang R Feng T. The vmPFC-IPL functional connectivity as the neural basis of future self-continuity impacted procrastination: the mediating role of anticipated positive outcomes. Behav Brain Funct. (2024) 20:11. doi: 10.1186/s12993-024-00236-z 38724963 PMC11083830

[19] Yang JJ Weng XC Guan LC Kuang PZ Zhang MZ Sun WJ . The role and mechanism of the medial temporal lobe system in perceptual priming. Acta Psychol Sin. (2003) 35:597–603.

[20] Wang XR Qiu LH. Research progress on gender differences in brain structural magnetic resonance imaging of adolescent depression. J Clin Radiol. (2023) 42:1689–92.

[21] Kang B Yoon DE Ryu Y Lee IS Chae Y. Beyond needling: integrating a bayesian brain model into acupuncture treatment. Brain Sci. (2025) 15:192. doi: 10.3390/brainsci15020192 40002525 PMC11852460

[22] Bai L Tian J Zhong C Xue T You Y Liu Z . Acupuncture modulates temporal neural responses in wide brain networks: evidence from fMRI study. Mol Pain. (2010) 6:73. doi: 10.1186/1744-8069-6-73 21044291 PMC2989943

[23] Deng D Liao H Duan G Liu Y He Q Liu H . Modulation of the default mode network in first-episode, drug-naïve major depressive disorder via acupuncture at Baihui (GV20) acupoint. Front Hum Neurosci. (2016) 10:230. doi: 10.3389/fnhum.2016.00230 27242492 PMC4869560

[24] Xu N He Y Wei YN Bai L Wang L. Possible antidepressant mechanism of acupuncture: targeting neuroplasticity. Front Neurosci. (2025) 19:1512073. doi: 10.3389/fnins.2025.1512073 40018358 PMC11865234

[25] Tamai H Komine S Kikuchi S Waki H. Exploring feasibility of fNIRS to assess delayed inhibition effect of prefrontal cortex for acute stress by acupuncture on GV20: a pilot study. Front Hum Neurosci. (2024) 18:1433312. doi: 10.3389/fnhum.2024.1433312 39619678 PMC11604711

[26] Xia R Ren J Wang M Wan Y Dai Y Li X . Effect of acupuncture on brain functional networks in patients with mild cognitive impairment: an activation likelihood estimation meta-analysis. Acupunct Med. (2023) 41:259–67. doi: 10.1177/09645284221146199 36790017

[27] Zhu XD. The impact of physical exercise on academic procrastination among college students: the mediating role of time management disposition. Sports Sci Technol. (2022) 5:140–2.

[28] Lin L Bai XW A. study on academic procrastination among college students based on the theory of planned behavior. Chin J Clin Psychol. (2014) 22:6–8.

[29] Feng HT Zheng WB. Study on exercise behavior of college students based on the theory of planned behavior. J Hebei Univ Sci Technol (Soc Sci). (2012) 12:7–9.

[30] Monds LA MacCann C Mullan BA Wong C Todd J Roberts RD. Can personality close the intention-behavior gap for healthy eating? An examination with the HEXACO personality traits. Psychol Health Med. (2016) 21:845–55. doi: 10.1080/13548506.2015.1112416 26584691

[31] Shukri M Jones F Conner M. Work Factors, Work-Family Conflict, the Theory of Planned Behaviour and Healthy Intentions: a Cross-Cultural Study. Stress Health. (2016) 32:559–68. doi: 10.1002/smi.2662 26643961

[32] Gong WQ. Study on influencing factors of exercise behavior among college students in Hunan Province based on TPB model [thesis]. Hunan Univ Sci Technol. (2023).

[33] Zhang W-J Xu M Feng Y-J Mao Z-X Yan Z-Y Fan T-F. The Value-Added Contribution of Exercise Commitment to College Students' Exercise Behavior: Application of Extended Model of Theory of Planned Behavior. Front Psychol. (2022) 13:869997. doi: 10.3389/fpsyg.2022.869997 35719512 PMC9204293

[34] Lin L. Intervention in procrastination: effects of the theory of planned behavior and implementation intention. Acta Psychol Sin. (2017) 49:13–5. doi: 10.3724/SP.J.1041.2017.00953

[35] Chen JM Lü Y. The effect of self-compassion on college students' procrastination: the serial mediating role of experiential avoidance and shame. Stud Psychol Behav. (2023) 21:109–23.

[36] Cao JQ Cao GK Zhang Y. Relationship between physical exercise and procrastination among college students: the mediating role of time management disposition. Adv Psychol Sci. (2018) 8:816–24.

[37] Qiu K. The relationship between physical activity and academic procrastination among college students: The mediating role of self-esteem [Doctoral dissertation]. Zhejiang Normal University (2023). doi: 10.27464/d.cnki.gzsfu.2023.001985

[38] Li B Tang H He G Jin Z He Y Huang P . Tai Chi enhances cognitive training effects on delaying cognitive decline in mild cognitive impairment. Alzheimers Dement : J Alzheimers Assoc. (2023) 19:136–49. doi: 10.1002/alz.12658 35290704

[39] Converse AK Ahlers EO Travers BG Davidson RJ. Tai chi training reduces self-report of inattention in healthy young adults. Front Hum Neurosci. (2014) 8:13. doi: 10.3389/fnhum.2014.00013 24478679 PMC3902356

[40] Wang Y Tian J Yang Q. Tai Chi exercise improves working memory capacity and emotion regulation ability. Front Psychol. (2023) 14:1047544. doi: 10.3389/fpsyg.2023.1047544 36874821 PMC9983368

[41] Ni YK Zhao JZ Li QL Guo TF Wang MH. Differences in intertemporal choice between high and low procrastinators: a study based on ERP. Stud Psychol Behav. (2019) 17:901–8.

[42] Guo R. Neural dynamics of anxiety regulation in monetary reward processing: evidence from ERPs. Adv Psychol. (2024) 14:1650–9. doi: 10.12677/ap.2024.1410726

[43] Xiao YX. P300 and cognitive processing: methods, mechanisms, and applications. China J Health Psychol. (2015) 23:1425–30.

[44] Demirayak P Kiyi I Işbitiren YÖ Yener G. Cognitive load associates prolonged P300 latency during target stimulus processing in individuals with mild cognitive impairment. Sci Rep. (2023) 13:15956. doi: 10.1038/s41598-023-43132-8 37743392 PMC10518304

[45] Shi XJ Zhang AZ Niu X Qu FB. Review on time dimensions affecting procrastination: based on time perspective. Adv Psychol. (2021) 11:1061–70.

[46] Wang NN. Research progress on procrastination in the time dimension. Soc Sci Front. (2020) 9:7–13.

[47] Shu LC. Uncertain Decision-Making Mechanism Based on Decision Neuroscience. Hangzhou: Zhejiang University. (2009).

[48] Xu H Gu L Zhang S Wu Y Wei X Wang C . N200 and P300 component changes in Parkinson's disease: a meta-analysis. Neurol Sci. (2022) 43:6719–30. doi: 10.1007/s10072-022-06348-6 35982362

[49] Zhou HB. N200 EEG component and Chinese word processing. J Contemp Educ Theory Prac. (2016) 8:58–60.

[50] Zhou HB. N200 EEG component and Chinese word processing. Contemp Educ Theory Prac. (2016) 8:60.

[51] Zhao XX Sun L Wang CM Wang EC Li H Wu ZL . Event-related potential study on working memory characteristics in adults with attention deficit hyperactivity disorder. Chin J Psych. (2020) 53:831–6.

[52] To PYL Lo BCY Ng TK Wong BPH Choi AWM. Striving to avoid inferiority and procrastination among university students: the mediating roles of stress and self-control. Int J Environ Res Public Health. (2021) 18:6491. doi: 10.3390/ijerph18115570 34071056 PMC8197152

[53] Xue LL Guan XJ editors. Revision of the general procrastination scale. Abstracts of the 10th National Congress of Psychology. (2005).

[54] Yang YJ Wu A Chen A Xie A Zhang A Bu A . Reliability and validity of the General Procrastination Scale in middle school students. Chin J Behav Med Brain Sci. (2024) 33:168–73.

[55] Yip MCW Chung OLL. Psychometric properties of the Chinese version of procrastination assessment scale for students. Front Psychol. (2022) 13:1016116. doi: 10.3389/fpsyg.2022.1016116 36275234 PMC9583896

[56] Hou Y Gai NC. Current status and causes of academic procrastination among college students. Psychol Res J. (2008) 1:91–6.

