# Correction: Effects of acupuncture and exercise on EEG characteristics and cognitive control in college students with high procrastination tendency

**DOI:** 10.3389/fpubh.2026.1852684

**Published:** 2026-04-22

**Authors:** Xinwang Chen, Yajing Guo, Ce Shi, Jing Wen, Yuan Gao, Lihua Wu, Haishui Jiang, Yongqi Yuan, Linze Wu, Huihui Yin, Yiming Wu

**Affiliations:** 1 College of Acupuncture-Moxibustion and Tuina, Henan University of Traditional Chinese Medicine, Zhengzhou, China; 2 College of the First Clinical Medicine, Anhui University of Chinese Medicine, Hefei, China; 3 College of Rehabilitation Medicine, Henan University of Traditional Chinese Medicine, Zhengzhou, China; 4 The Third Affiliated Hospital, Henan University of Traditional Chinese Medicine, Zhengzhou, China

**Keywords:** acupuncture, cognitive function, event-related potential, procrastination, university students

The funder “Henan Provincial Science and Technology Research and Development Program Joint Fund: Neurodynamic Characteristics of Acupuncture on Patients with Insomnia Complicated with Cognitive Impairment Based on EEG Power Spectrum and Spindle-Slow Wave Coupling”, Grant Number 242301420085 to Xinwang Chen was erroneously omitted.

The funder “Special Project for Traditional Chinese Medicine Scientific Research of Henan Province: Randomized Controlled Study on the Clinical Effect of Abdominal Massage in Patients with Generalized Anxiety Disorder and Related Events”, Grant number 2022ZYZD12 to Yiming Wu were erroneously omitted.

The corrected Funding statement appears below:

“The author(s) declared that financial support was received for this work and/or its publication. The proposed experiments will be supported by the Double First-Class Discipline Construction Project of Henan University of Traditional Chinese Medicine (No. HSRP-DFCTCM-T-10). This work was supported by Henan Provincial Science and Technology Research and Development Program Joint Fund: Neurodynamic Characteristics of Acupuncture on Patients with Insomnia Complicated with Cognitive Impairment Based on EEG Power Spectrum and Spindle-Slow Wave Coupling, Grant Number 242301420085 to Xinwang Chen; and by Special Project for Traditional Chinese Medicine Scientific Research of Henan Province: Randomized Controlled Study on the

Clinical Effect of Abdominal Massage in Patients with Generalized Anxiety Disorder and Related Events, Grant number 2022ZYZD12 to Yiming Wu. The sponsor and funder are not involved in study design, data collection and management, data analysis and data interpretation, report writing, and publication decisions or manuscript preparation, and has no final authority over any of these activities.”

The original version of this article has been updated.

