# Supplementary material for: Effects of acupuncture and exercise on EEG characteristics and cognitive control in college students with high procrastination tendency
Source: Front Public Health. 2026 Apr 2;14:1725633. doi: 10.3389/fpubh.2026.1725633 (PMC13085052; doi:10.3389/fpubh.2026.1725633)
Supplement: Supplementary file 1 [file Supplementary_file_1.docx]

Informed Consent Informed Notification page

Introduction to the study

1. Why is this study being conducted?

Procrastination, defined as "the act of voluntarily postponing a planned task when the consequences can be expected to be harmful", has become a prevalent behavioral disorder worldwide. Research shows that procrastination is particularly common among college students, more than 70% of students admit that they have academic procrastination, of which serious academic procrastination accounts for about 16%, a survey of 22,896 college students shows that more than ninety percent of college students have procrastination habits, of which more than sixty percent are mainly reflected in learning, in addition to academics, college students also have procrastination behaviors in daily life, such as delaying the completion of daily tasks, delaying decision-making, etc. Procrastination manifests itself in task postponement, indirect avoidance, inefficiency, emotional distress, physical symptoms, and decreased self-confidence. For example, they often put off the date of completing the task again and again, always wait until the last minute to start the action on the grounds of "not urgent" or "waiting for inspiration", give priority to unimportant things (such as scrolling through mobile phones, playing games) to avoid work or study that require concentration, even if they have already started the task, they will be frequently distracted, resulting in a lot of time but not a high degree of completion, because the task is not completed on time, the procrastinator will repeatedly experience self-blame and guilt, and then form a vicious circle. Procrastinating on important tasks for long periods of time can weaken self-efficacy, doubt one's abilities, and even avoid taking on challenging tasks. Procrastination not only affects an individual's learning and work efficiency, but can also lead to an increase in psychological stress and a decrease in the quality of life. The persistence of such behaviour not only leads to a loss of individual productivity, but also a significant socio-economic burden – the World Health Organization estimates that procrastination costs more than $1.2 trillion annually globally.

The purpose of this study was to observe the ERP characteristics of procrastination population, evaluate the clinical efficacy of acupuncture and exercise intervention on the cognitive function of procrastination, and explore the ERP changes of the effect of acupuncture and exercise intervention on cognitive function of procrastination population.

This study will be conducted in the Third Affiliated Hospital of Henan University of Traditional Chinese Medicine, and more than 90 subjects are expected to voluntarily participate.

2. Who is suitable to participate in the study?

The inclusion criteria for the study population were:

(1) Full-time college students;

(2) The total score of the Reye's General Procrastination Scale ≥ 70 points;

(3) Have not taken psychotropic drugs or received related acupuncture treatment in the past 30 days;

(4) Those who voluntarily participate in clinical research and agree to sign the informed consent form.

Exclusion Criteria:

(1) Organic mental disorder, or illness related to surgical history or psychoactive substances;

(2) Have a family history of other mental illnesses such as dementia, schizophrenia, mania, addiction, etc.;

(3) Subjects with impaired consciousness or agitation who cannot cooperate with the completion of paradigm tasks and EEG collection;

(4) Patients with skin lesions or skin diseases.

3. What you will need to do if you participate in the study

1.Before you are enrolled in the study, you will undergo the following tests to determine if you can participate in the study:

Your doctor will ask about you, take a medical history and do a physical exam. You need to cooperate with the doctor to complete the questionnaire and the corresponding EEG examination, and go to the hospital on time every week for outpatient treatment. If you are an eligible incorporator, you can voluntarily participate in the study and sign an informed consent form.

2. If you pass the above tests, you will be studied as follows. At the beginning of the study, you will be given acupuncture or control treatment based on random numbers provided by the computer, and the patients participating in this study may be divided into acupuncture, running, and tai chi groups, and neither you nor your doctor will be able to know and choose any treatment in advance, and the treatment observation will last for 4 weeks.

Acupuncture group: acupuncture treatment was carried out according to the proposed acupuncture treatment plan; Running group: Treatment was carried out according to the proposed treatment plan for the running group. Tai Chi group: Treat according to the proposed treatment plan of Tai Chi group.

Before treatment: You should go to the hospital and truthfully report the changes in your condition to the doctor, who will collect your medical history and physical examination results, and give you a brain function evaluation and scale evaluation.

Week 4 post-treatment: At this time, the study ends. You should go to the hospital, where the doctor will ask you to record the changes in your condition and give you a brain function assessment and a scale evaluation.

3. Other matters that require your cooperation

You'll need to come to the hospital for the follow-up time agreed between your doctor and you. Your follow-up is very important because your doctor will judge if the research measures you received are really working.

You cannot use psychiatric-related medications or receive acupuncture treatment for psychiatric disorders during the study. If you need other treatments, please contact your doctor beforehand.

regulations on diet, living and living; Eat less spicy and greasy food.

4. Anticipated circumstances and/or reasons why your participation in the trial may be terminated

A very small number of people may have adverse reactions such as dizziness, pain, and subcutaneous hematoma during the experiment, or even lead to the termination of the trial. The reason for this is related to the individual's response to acupuncture treatment; The investigator did not follow the treatment protocol during the treatment period; The subject no longer cooperates with the investigator's arrangement due to personal reasons, and the investigator believes that the patient's compliance is poor and is not suitable for the study. If there is a treatment-related situation that leads to the termination of the trial, we promise to do our best to provide the best possible treatment.

Fourth, the possible benefits of participating in the study

You and society may benefit from this study. You will receive 4 weeks of acupuncture treatment, 3 brain function evaluations and scale evaluations, and the subject may experience some improvement in procrastination symptoms and brain function, including the possibility that this study may help develop a new treatment regimen for other patients with similar conditions.

You will receive good medical care for the duration of the study.

5. Possible adverse reactions, risks, discomfort and inconvenience of participating in the study

All treatments have the potential for side effects. The acupuncture treatments used in this study are all safe methods, and you may feel soreness, numbness, heaviness, and swelling during the acupuncture process, which are normal symptoms of acupuncture. There may be adverse reactions after acupuncture, but they are few and mild, and you may faint due to your physical problems or emotional stress during acupuncture, which can be relieved by stopping acupuncture and appropriate rest; Bleeding and hematoma may occur after acupuncture, which may disappear with local compression: but if there is infection at the acupuncture site, it should be treated by a doctor promptly.

If you experience any discomfort during the study, or if there is a new change in your condition, or any unexpected situation, whether it is related to the treatment or not, you should promptly inform your doctor and he/she will make a judgment and medical treatment. The physician (or investigator) will do his best to prevent and treat possible injuries due to this study.

You'll need to be in the hospital for regular follow-up appointments during the study, which can be a nuisance or inconvenience for you.

EEG: Symptoms of allergy to conductive creams may occur during EEG, which is manifested as rashes, rashes, itching, swelling, etc., but they are very rare.

In addition, acupuncture interventions may not be effective, and the disease may continue to progress due to ineffective treatment or comorbidities. During the study, if the physician finds that the acupuncture intervention used in this study is ineffective, the study will be discontinued and replaced with another treatment that may be effective.

6. Related fees

In order to compensate for the inconvenience that you may cause to participate in this study, this study will pay for the study-related examinations (brain function evaluation and scale evaluation before treatment, brain function evaluation and scale evaluation after 2 or 4 weeks of treatment) during your participation in this study, and provide acupuncture treatment free of charge, and the study will not increase your additional cost. If you withdraw from the study, you will not be reimbursed for subsequent examinations. In the event of test-related damages, the research team will pay your medical expenses and provide corresponding financial compensation in accordance with laws and regulations.

If you have treatment and examinations required for other diseases at the same time, the examination fee and medical treatment fee will not be included in the scope of free of charge.

If you combine the treatments and tests required for other conditions, and the cost of switching to other treatments because the treatment is ineffective, it will not be covered by the free of charge.

7. Is personal information confidential?

Your medical records (study records/CRFs, lab sheets, etc.) will be kept intact at the hospital and the doctor will record the results of the lab tests on your outpatient medical records. The investigator, sponsor representative, ethics committee, and study oversight will be allowed access to your medical records. Any public report on the results of this study will not reveal your personal identity. We will make every effort to protect the privacy of your personal medical information to the extent permitted by law.

In addition to this study, it is possible that your medical records and pathology specimens will be reused in future studies. You may also now declare that you will refuse to utilize your medical records and pathology specimens for studies other than this one.

8. How can I get more information?

You can ask any questions about this study at any time. Your doctor will leave you his/her phone number so that he or she can answer your questions.

If you have any complaints about participating in the study, please contact the Office of the Ethics Committee.

Your doctor will keep you informed if there is any important new information during the course of the study that may affect your desire to continue participating in the study.

9. You can voluntarily choose to participate in the research and withdraw from the research halfway

Whether or not to participate in the study is entirely up to your voluntariness. You may refuse to participate in the study, or withdraw from the study at any time during the course of the study, without affecting your relationship with your physician or with any loss of medical or other benefits to you.

Your physician or investigator may discontinue your participation in this study at any time in your best interests.

If you do not participate in this study, or drop out of the study, there are many other alternative treatments that you do not have to choose to participate in this study in order to treat your condition.

If you withdraw from the study for any reason, you may be asked about your use of the trial drug. You may also be asked to have lab tests and a physical exam if your doctor thinks so. This is good for your health.

10. What should I do now?

It's up to you to decide whether or not to participate in this study. You can discuss it with your family or friends before making a decision.

Before you make a decision to participate in the study, ask your doctor as many questions as possible until you fully understand the study.

Thank you for reading the above material. If you decide to participate in this study, please tell your doctor or research assistant and he/she will arrange everything for you.

Please keep this information.

Informed Consent Form and Consent Signature Page

Subject Consent Statement

I have read the above presentation of this study and have had the opportunity to discuss and ask questions about this study with my doctor. All the questions I asked were answered satisfactorily.

I am aware of the risks and benefits that may arise from participating in this study. I understand that participation in the study is voluntary, I confirm that I have had sufficient time to consider this, and I understand that:

- I can always ask my doctor for more information.
- I can withdraw from this study at any time without discrimination or retaliation, and my medical treatment and rights will not be affected.

I also know that if I withdraw from the study, especially if I withdraw from the study due to medication, it will be very beneficial for me and the whole study if I tell the doctor about the change in my condition and complete the corresponding physical examination and physical and chemical examination.

If I need to take any other medication due to a change in my condition, I will seek the doctor's advice beforehand or tell the doctor truthfully afterwards.

I consent to access to my research materials by the study regulatory authority, the ethics committee, or the sponsor's representative.

I will be given a signed and dated copy of the informed consent form.

In the end, I decided to agree to participate in this study.

Subject Signature: Date: _ _ _ _ _

Subject's contact number: Mobile phone number:

Signature of the subject's legal guardian: Date: _ _ _ _ _

Legal Guardian Contact Number: Mobile Phone Number:

Investigator's Statement

I confirm that I have explained the details of this trial, including its rights and possible benefits and risks, to the subject and give them a copy of the signed informed consent form.

Investigator Signature: Date: _ _ _ _ _

Investigator's Work Phone: Mobile phone number:
